# Phylogenetic Analysis and In Vitro Bifunctional Nuclease Assay of *Arabidopsis* BBD1 and BBD2

**DOI:** 10.3390/molecules25092169

**Published:** 2020-05-06

**Authors:** A. K. M. Mahmudul Huque, Won Mi So, Min Kyoung You, Jeong Sheop Shin

**Affiliations:** 1 Division of Life Sciences, Korea University, Seoul 02841, Korea; mahmudornob@korea.ac.kr (A.K.M.M.H.); thso1124@gmail.com (W.M.S.); 2 Department of Genetic Engineering and Graduate School of Biotechnology, College of Life Sciences, Kyung Hee University, Yongin 17104, Korea

**Keywords:** *Arabidopsis*, DUF151, nuclease, OmBBD, AtBBD1

## Abstract

Nucleases are a very diverse group of enzymes that play important roles in many crucial physiological processes in plants. We previously reported that the highly conserved region (HCR), domain of unknown function 151 (DUF151) and UV responsive (UVR) domain-containing OmBBD is a novel nuclease that does not share homology with other well-studied plant nucleases. Here, we report that DUF151 domain-containing proteins are present in bacteria, archaea and only Viridiplantae kingdom of eukarya, but not in any other eukaryotes. Two *Arabidopsis* homologs of OmBBD, AtBBD1 and AtBBD2, shared 43.69% and 44.38% sequence identity and contained all three distinct domains of OmBBD. We confirmed that the recombinant MBP-AtBBD1 and MBP-AtBBD2 exhibited non-substrate-specific DNase and RNase activity, like OmBBD. We also found that a metal cofactor is not necessarily required for DNase activity of AtBBD1 and AtBBD2, but their activities were much enhanced in the presence of Mg^2+^ or Mn^2+^. Using a yeast two-hybrid assay, we found that AtBBD1 and AtBBD2 each form a homodimer but not a heterodimer and that the HCR domain is possibly crucial for dimerization.

## 1. Introduction

Nucleases that hydrolyze the phosphodiester linkages of nucleic acids comprise the largest number of different structures and mediate a variety of biological functions [1]. Nucleases function in various key metabolic processes, such as DNA restriction enzyme digestion, site-specific recombination, DNA repair, pre-mRNA splicing and RNA interference as well as DNA replication and RNA processing. In plants, nucleases play important roles in many crucial physiological processes, such as senescence [2,3,4,5], programmed cell death [6,7,8,9], xylem development [10,11], flower and pollen development [12,13] as well as biotic [14,15] and abiotic [16,17] stress responses. Nucleases are categorized into exonucleases and endonucleases based on the enzymatic properties [16]. Exonucleases break the phosphodiester bonds sequentially from one end of the nucleic acid. In *Arabidopsis*, Werner syndrome-like exonuclease (WEX) and defective in pollen organelle DNA degradation1 (DPD1) exonucleases that require Mg^2+^ ion for their nuclease activity have been reported [12,18] and the latter is involved in pollen development. The endonucleases are classified into two major classes based on their cofactors and pH dependency [19]: Zn^2+^-dependent endonucleases that exhibit optimum activity with acidic pH, and Ca^2+^-dependent endonucleases that require neutral pH [19]. Several Ca^2+^-dependent nucleases can also show considerable activity with Mg^2+^, Mn^2+^, or Co^2+^ [20,21,22].

In plants, endonucleases can also be sub-categorized into S1 nuclease-like endonuclease and staphylococcal-like endonuclease according to their similarity to the bacterial endonucleases [23]. In *Arabidopsis*, five S1-like endonucleases (ENDO1 to ENDO5), having distinctive S1/P1 nuclease domain (Pfam PF02265), have been identified and biochemically characterized [24,25]. Of these, ENDO1 (also known as AtBFN1) is an endoplasmic reticulum-localized bifunctional endonuclease (having both DNase and RNase activities), which requires Zn^2+^ and slightly basic pH for optimum activity [25]. ENDO1 recognizes and cleaves all types of mismatches with a great efficiency [24] and is possibly associated with leaf and stem senescence [4]. Likewise, staphylococcal-like endonucleases are also very common in plant tissues. For example, *Arabidopsis* AtCAN1 and AtCAN2 are plasma membrane-localized staphylococcal-like nucleases that include a characteristic SNase domain (Pfam PF00565) and require a Ca^2+^ ion to hydrolyze ssDNA, dsDNA, and RNA as well [26,27,28]. AtCAN2 is a negative regulator of salt stress response [16].

We previously reported that OmBBD (*Oryza minuta* bifunctional nuclease in basal defense response), identified from a wild species of rice (*Oryza minuta*), is a novel bifunctional nuclease that neither contains any distinctive S1/P1 nuclease and SNase nuclease domains nor shares any conserved residues with those of the well-studied S1-like and staphylococcal-like endonucleases [15]. OmBBD contains three functional domains, a highly conserved region (HCR) domain for protein–protein interaction [29], a domain of unknown function 151 (DUF151) that is also known as either bifunctional nuclease (BFN) domain (IPR003729 in the InterPro database) or DNase-RNase domain (PF02577 in the Pfam database), and a UV responsive (UVR) domain for protein–protein interaction [30] or protein-DNA interaction [29]. Seo et al. (2013) [29] reported that two *Arabidopsis* homologs of OmBBD, AtBBD1 and AtBBD2, specifically bind to a jasmonic acid responsive element (JARE) (G/CTCCTGA) of *AtJMT* promoter and repress its expression through histone deacetylation upon MeJA treatment.

In this study, we analyzed the phylogenetic relationships of 3234 proteins containing the DUF151 domain that is thought to play an important role in bifunctional nuclease activity of OmBBD. We found that AtBBD1 and AtBBD2 obviously contain all three distinctively conserved domains of OmBBD, and we speculated that AtBBD1 and AtBBD2 somehow should function as DNase-RNase bifunctional nucleases despite also acting as transcriptional repressors. Thus, we purified the recombinant AtBBD1 and AtBBD2 and characterized their bifunctional nuclease activity in vitro. We also examined their enzymatic activity according to metal ions, temperature and pH.

## 2. Results and Discussion

### 2.1. DUF151-Containing Proteins Are Phylogenetically Divided into Two Major Groups, and Eukaryotic Proteins Are Only Identified in the Viridiplantae

**Figure 1:**
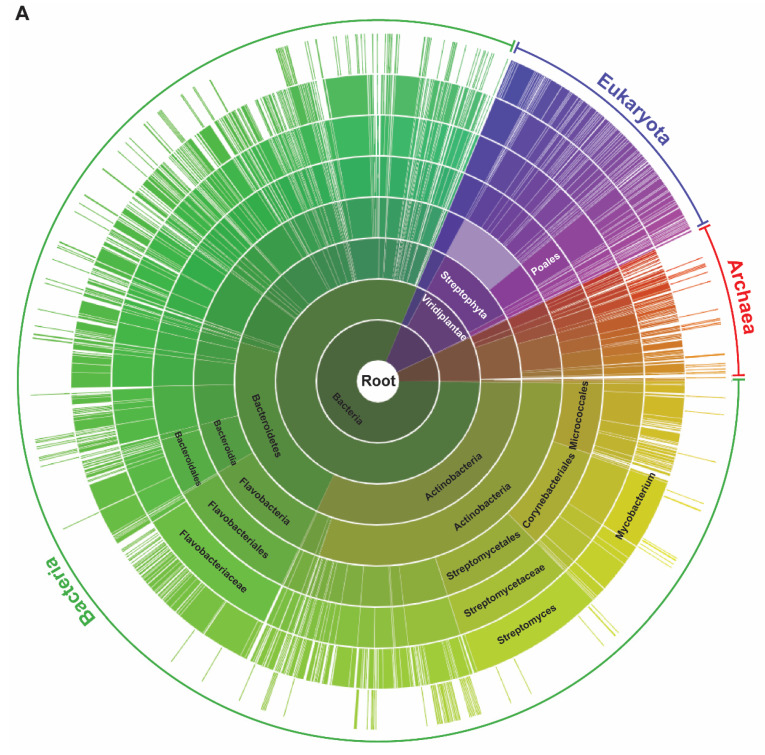

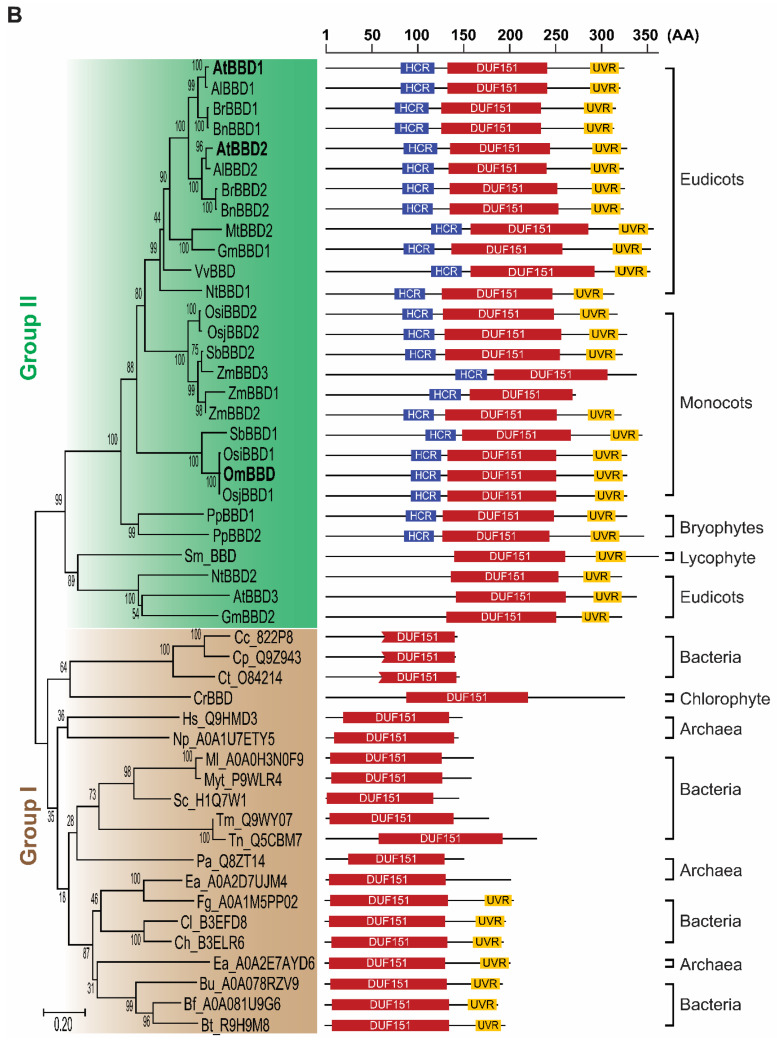
Analysis of DUF151 domain-containing proteins. (**A**) Sunburst visualization of the distribution of 3234 DUF151 domain-containing proteins existing in three domains, Bacteria, Archaea and Eukarya, obtained from the Pfam database. (**B**) Phylogenetic analysis of 48 representative BBD-like proteins. The detailed information about proteins was presented in Table S1. The names of plant proteins were designated as BBD preceded by the initial of genus and species name, while the proteins of unicellular organisms were named as described in the Uniprot ID. The phylogenetic tree was constructed using the neighbor-joining method in the MEGA-X program. The pairwise deletion option was applied to avoid gaps and/or missing data. The reliability of different phylogenetic clusters was evaluated by the bootstrap test (500 bootstrap replications).

To investigate the evolutionary features of BBD proteins, a sunburst diagram was initially constructed with 3234 proteins that contain the DUF151 domain identified in the Pfam database (Figure 1A). We found that about 80% of the DUF151-containing proteins are of bacterial origin and 13% and 6% are, respectively, of Viridiplantae (“green plant”) and archaea, but none were identified from other eukaryotic organisms. These results imply that this protein family evolved from a common ancestor conservatively from bacteria to plant, but for some reason, the DUF151 domain did not exist in any eukaryotes other than Viridiplantae clade. The Rv1829 of *Mycobacterium tuberculosis*, which is a protein comprised entirely of DUF151 domain, is critical for its in vivo survival and virulence, as it confers resistance to host macrophage-derived carbon monoxide toxicity [31], indicating that DUF151 domain-containing proteins could have important biological functions in other organisms. However, the exact molecular function of this ancient and conserved DUF151 domain remains unknown.

Next, we constructed a phylogenetic tree with the domain structures using 48 representative DUF151 domain-containing proteins selected from bacteria, archaea and Viridiplantae: 14 from bacteria, 5 from archaea, 15 from eudicots, 10 from monocots, 2 from bryophytes, 1 from lycophyte and 1 from unicellular algae (*Chlamydomonas reinhardtii*) (Figure 1B). Detailed information about the proteins is presented in Table S1. Our phylogenetic tree clearly divided the DUF151-containing proteins into two major groups; one solely with unicellular organisms including bacteria, archaea and algae (light brown), named as Group I, and the other with multicellular eukaryotes including bryophytes, lycophytes and angiosperms (green), named as Group II (left panel in Figure 1B). The protein domain structure showed several distinct patterns (right panel in Figure 1B). None of the Group I proteins had an extended N-terminal region containing a HCR domain, so their sizes are relatively shorter than those of Group II proteins. The DUF151 domain existed alone or together with an HCR and/or a UVR domain. However, the HCR domain was observed only in plant BBDs, except for a lycophyte protein, SmBBD, and three eudicot proteins, NtBBD2, AtBBD3 and GmBBD2, and thereby the latter three grouped into a separate clade away from other eudicots. These results imply that a UVR domain was incorporated into DUF151-containing proteins in bacteria and then an HCR domain appeared later into higher plants during the process of evolution. The HCR domains of *Arabidopsis* AtBBD1 and AtBBD2 have been reported to interact with AtJAZ1 and AtJAZ4, which repress the activity of transcription factors that execute responses to JA-Ile [29]. Thus, the presence of the HCR domain in the DUF151 domain-containing proteins in higher plants could play different and/or additional molecular functions compared to the unicellular organisms.

### 2.2. AtBBD1 and AtBBD2 Contain Three Characteristic Domains and Are the Closest Arabidopsis Homologs of OmBBD

**Figure 2:**
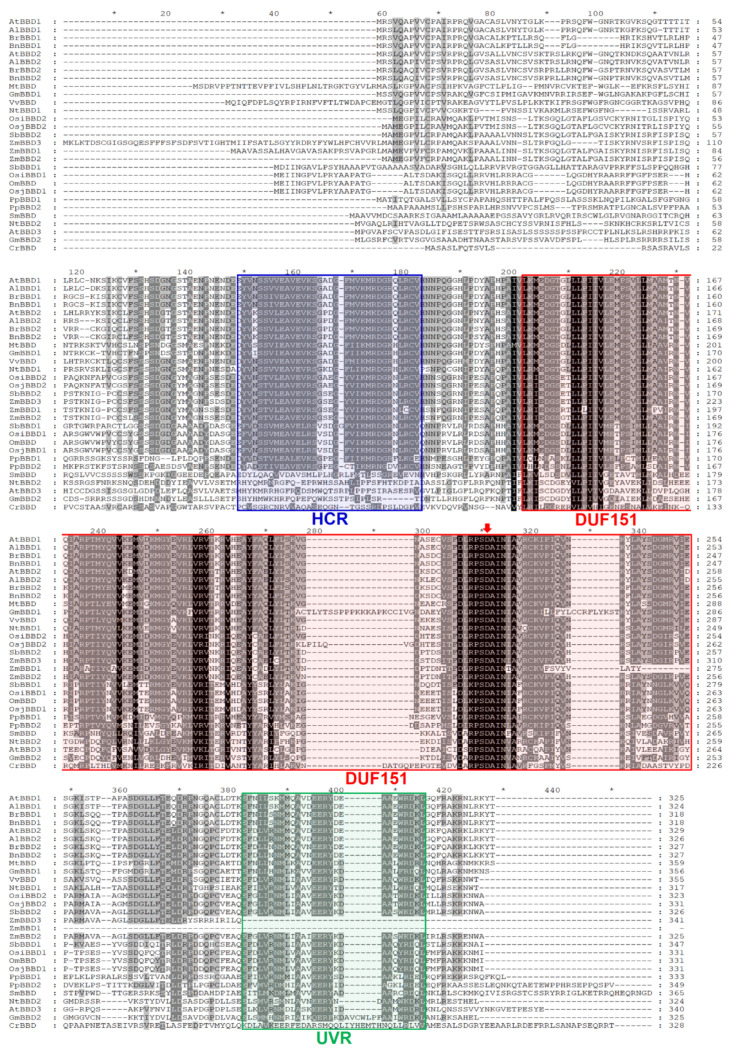
Amino acid sequence alignment of 29 eukaryotic BBD-like proteins from Viridiplantae kingdom. Identical residues are highlighted in black, and similar residues are highlighted in gray. The blue box, red box and green box represent the Highly Conserved Region (HCR), Domain of Unknown Function 151 (DUF151) and UVR domains, respectively. Gaps are inserted to maximize homology. The red arrow indicates aspartic residue within the predicted active site of DUF151 domain.

Next, a multiple alignment of amino acid sequences of 29 proteins belonging to the Viridiplantae was assembled (Figure 2). We found that two *Oryza sativa* BBD1s, from subspecies indica (OsiBBD1) and japonica (OsjBBD1), had more than 99% sequence similarity with OmBBD, implying that these proteins may also have a similar molecular function and physiological response. Of these DUF151-containing proteins, *Arabidopsis* homologs of OmBBD were of particular interest. OmBBD is known as a cofactor-independent novel bifunctional nuclease, which does not share any conserved domains responsible for nuclease activities of well-known S1-like and staphylococcal-like endonucleases [15]. Of three *Arabidopsis* homologs identified, AtBBD1 and AtBBD2 shared 43.69% and 44.38% amino acid sequence identity with OmBBD, respectively, and contained all three conserved characteristic domains. But AtBBD1 and AtBBD2 showed a very high sequence identity of 80.31%, indicating their functional redundancy in the genome. The lower sequence identity among OmBBD, AtBBD1 and AtBBD2 mainly appeared in the highly variable N-terminal regions (Figure 2). However, AtBBD3 belonged to a distant clade due to its absence of the HCR domain and lower similarity with AtBBD1 and AtBBD2. Because AtBBD1 and AtBBD2 share the same pattern of conserved domains with OmBBD, we hypothesized that AtBBD1 and AtBBD2 also possess DNase and RNase activities as OmBBD does.

### 2.3. Recombinant MBP-Tagged AtBBD1 and AtBBD2 Proteins Purified Using Amylose Affinity Chromatography

**Figure 3:**
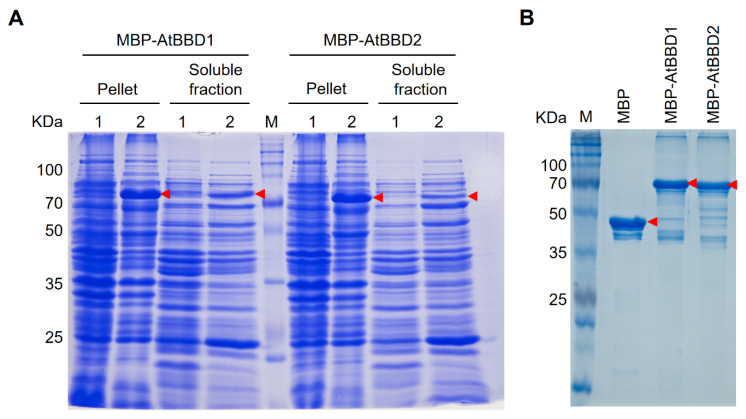
SDS-PAGE gels of MBP, AtBBD1 and AtBBD2 Proteins. (**A**) SDS-PAGE of MBP-BBD1 and MBP-BBD2 proteins in pellets and soluble fractions. Total proteins were electrophoresed on a 12% SDS-PAGE gel and stained with Coomassie blue R-250. Lane M, protein marker; 1, uninduced cells; 2, induced cells. Target proteins are indicated by red arrowheads. (**B**) SDS-PAGE of the purified MBP, MBP-BBD1 and MBP-BBD2 proteins. Purified proteins were electrophoresed on a 12% SDS-PAGE gel and stained with Coomassie blue R-250. Purified target proteins are indicated by red arrowheads. Lanes M, protein marker; 1, purified MBP; 2, purified MBP-BBD1; 3, purified MBP-BBD2.

Unlike OmBBD, the purification of AtBBD1 and AtBBD2 proteins with low molecular weight His and GST tags were particularly challenging due to their high insolubility (Figure S1A,B). We also tried to use the non-denaturing detergent *N*-Lauroylsarcosine sodium salt followed by an exchange of detergent with Triton X-100 [32], a combination of Trixon X-100 and CHAPS [33], or Brij 35 [34] according to earlier reports for purifying biologically active insoluble proteins from inclusion bodies. We failed to purify them, even though the solubility of our target proteins increased (Figure S2A–D). Instead, we purified them with the large molecular weight MBP tag that helps solubilize the insoluble proteins. The MBP-tagged AtBBD1 and AtBBD2 proteins were induced well in the presence of IPTG and the soluble fraction contained a significant amount of recombinant proteins (Figure 3A). Then, we purified the recombinant MBP-AtBBD1 and MBP-AtBBD2 proteins using amylose affinity chromatography. The expected molecular weights of MBP-AtBBD1 and MBP-AtBBD2 were 79.11 KDa and 79.99 KDa, respectively. We found that the top band with the strongest intensity in a gel matches the expected target size (Figure 3B). Although several off-target protein bands appeared with our target, their overall intensities were negligible.

### 2.4. AtBBD1 and AtBBD2 Possess Non-Substrate-Specific DNase Activity that Is Affected by Temperature and pH But Do Not Require Bivalent Cations

**Figure 4:**
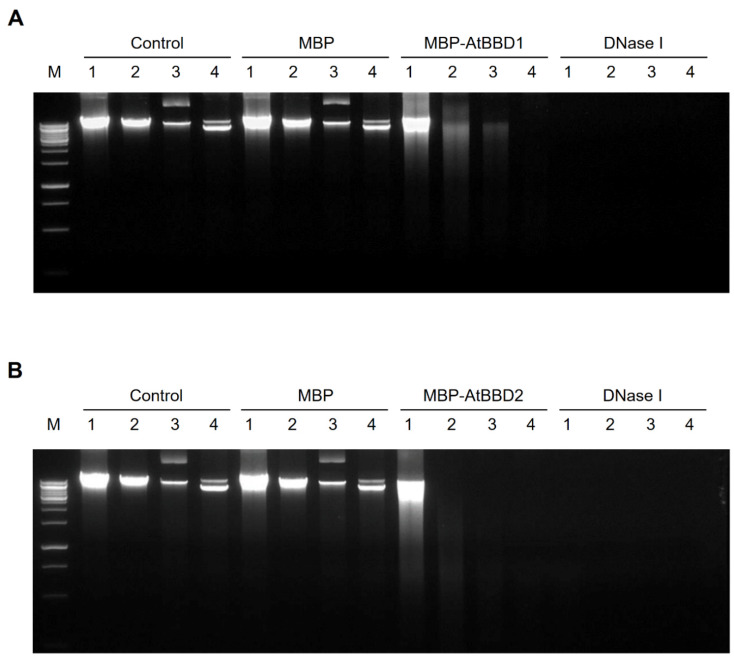
DNase activity assay of purified MBP-AtBBD1 and MBP-AtBBD2 proteins (**A**,**B**). The mock (without any protein) and the purified MBP protein were used as negative controls. (**A**) DNase activity of MBP-AtBBD1. DNA (0.5 to 1 μg) was incubated with 1 μg of purified protein at 37 °C for 3 h. The degraded DNA products were loaded onto a 1.0% agarose gel. (**B**) DNase activity of MBP-AtBBD2. DNA (0.5 to 1 μg) was incubated with 1 μg of purified protein at 37 °C for 3 h. The degraded DNA products were loaded onto a 1.0% agarose gel. Lanes M, DNA marker; 1, salmon sperm DNA; 2, *Arabidopsis* genomic DNA; 3, linearized plasmid DNA; 4, intact circular plasmid DNA. One unit of commercial DNase I was used as a positive control.

For in vitro DNase assay of AtBBD1 and AtBBD2, we used salmon sperm DNA, *Arabidopsis* genomic DNA, intact circular plasmids, and digested linear plasmids as substrates. The smearing or the disappearance of all four substrate DNAs by MBP-AtBBD1 and MBP-AtBBD2 but not by MBP (a negative control) indicated that both AtBBD1 and AtBBD2 possess non-substrate-specific DNase activity (Figure 4A,B). Moreover, the degradation of intact circular plasmids implied that these proteins are endonucleases, whereas DNase I that was used as a positive control completely digested target DNA because of its highly efficient exonuclease activity. In addition, because we did not use any bivalent cations as cofactors, it is certain that a cofactor is not necessarily required for the DNase activity of AtBBD1 and AtBBD2.

**Figure 5:**
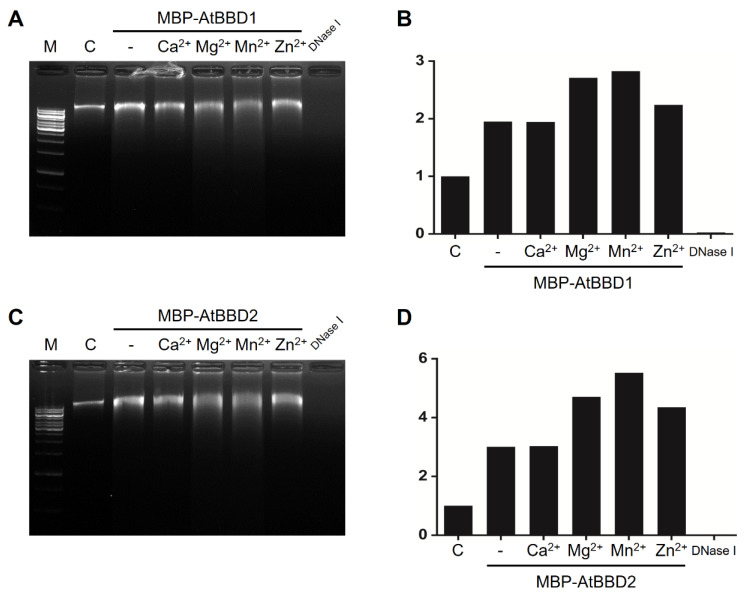
Effect of bivalent cations on DNase activity of MBP-AtBBD1 and MBP-AtBBD2. “C” denotes control, meaning without any proteins and cations. (**A**) MBP-AtBBD1 was incubated with *Arabidopsis* genomic DNA in the presence of 10 mM of CaCl_2_, MgCl_2_, MnCl_2_ and ZnCl_2_ at 37 °C for 1 h and visualized on a 1% agarose gel. (**B**) Quantification of the extent of *Arabidopsis* genomic DNA degradation showed in panel A. (**C**) MBP-AtBBD2 was incubated with *Arabidopsis* genomic DNA in the presence of 10 mM of CaCl_2_, MgCl_2_, MnCl_2_ and ZnCl_2_ at 37 °C for 1 h and visualized on a 1% agarose gel. (**D**) Quantification of the extent of *Arabidopsis* genomic DNA degradation showed in panel C. Band intensity of the degraded *Arabidopsis* genomic DNA in each lane of panel B and D was determined. The non-degraded DNA band intensity in the control lane (no enzyme) was set at 1.0 and then compared it to measure the relative value of degradation of DNA substrate in other lanes.

**Figure 6:**
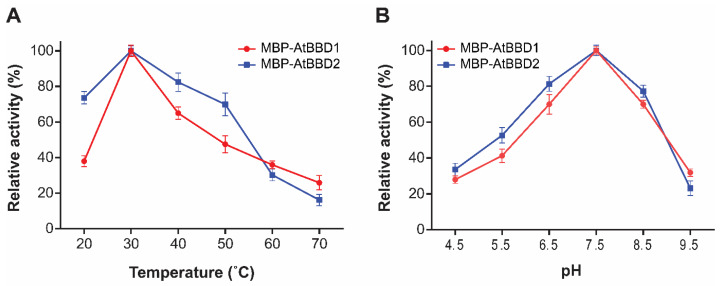
Effect of temperature and pH on the DNase activity of MBP-BBD1 and MBP-BBD2 proteins. (**A**) Temperature effect on DNase activity (Kunitz assay). The calculated specific activity values from Kunitz assay at various temperatures (20, 30, 40, 50, 60 and 70 °C) were plotted. (**B**) pH effect on DNase activity (Kunitz assay). The calculated specific activity values from Kunitz assay at various pH (4.5, 5.5, 6.5, 7.5, 8.5 and 9.5) were plotted. Each point represents the mean of triplicate assays and the error bars represents the standard deviations.

To determine whether divalent cations affect AtBBD1 and AtBBD2 DNase activity, we performed a DNase assay in the presence of 10-mM CaCl_2_, MgCl_2_, MnCl_2_, or ZnCl_2_ (Figure 5A,B). We observed that DNase activities of both AtBBD1 and AtBBD2 did not necessarily require any divalent metal cofactors, but the activity increased in the presence of Mg^2+^ or Mn^2+^ (Figure 5A,C). We quantified the extent of DNA degradation in the gels in the presence of metal ions (Figure 5B,D). We set the non-degraded DNA band intensity in the control lane (no enzyme) at 1.0 and then compared it to measure the relative value of degradation of DNA substrate in other lanes. We found that the relative value of DNA degradation by AtBBD1 without a metal ion was 1.95 but it increased to 2.71 and 2.82 in the presence of Mg^2+^ and Mn^2+^, respectively. Similarly, in the case of AtBBD2, we observed that its relative value of DNA degradation in the absence of a metal ion was around 3.0 but increased to 5.5 and 4.3 in the presence of Mg^2+^ and Mn^2+^, respectively. Enhanced DNase activity in the presence of Mn^2+^ is consistent with the property of OmBBD [15]. To investigate whether the DNase activity of AtBBD1 and AtBBD2 is dependent on temperature, we assayed DNase activity at several different temperatures. We found that AtBBD1 and AtBBD2 remained active in the range of temperature 20~70 °C and revealed the maximum activity at 30 °C. The DNase activity decreased sharply when it was above or below the optimum temperature (Figure 6A). We also examined the effect of pH on DNase activity and observed that the maximum DNase activity of both enzymes was shown at pH 7.5 (Figure 6B). A higher or lower pH than the optimum decreased the DNase activity. For example, the DNase activity plummeted to less than 10% in the extremely basic condition (pH 9.5). The optimal pH of an enzyme can provide us an insight on its subcellular localization. Most nucleases with optimal pH near neutral localize to the cytoplasm (pH 7.3) and nucleus (pH 7.2) and function in these organelles [35]. The neutral pH requirement of AtBBD1 coincides with its nuclear localization as reported earlier [29]. Taken together, we suggest that AtBBD1 and AtBBD2 possess DNase activities like OmBBD and that their activities are non-substrate-specific and do not require metal ions as cofactor but are enhanced by Mg^2+^ or Mn^2+^.

On the other hand, Seo el al. [29] reported that AtBBD1 and AtBBD2 act as transacting factors that redundantly and sequence specifically bind to the jasmonic acid responsive element (JARE) of the *AtJMT* promoter and transcriptionally repress its expression through histone deacetylation upon MeJA treatment. Their results of DNA binding ability and our findings of bifunctional nuclease activity on these two proteins are contradictory. It is not clear whether the reason for this contradiction is because Seo et al. used the soluble crude extracts of recombinant proteins for their experiments or if it is because AtBBD1 and AtBBD2 have a dual functionality—both bifunctional nuclease activity and transcriptional repression activity. The dual or multi-functionality of some proteins has been reported. For example, tomato multifunctional nuclease 1 (TBN1) is a Zn^2+^-dependent S1/P1 nuclease that cleaves DNA and RNA in both double- and single-stranded forms and plays multiple cellular functions including apoptosis, tissue differentiation, vascular system development and stress response [36]. However, Koval et al. [37] reported that TBN1 has another novel phospholipase activity related to the apoptosis process. In humans, phospholipid scramblase 1 (hPLSCR1) is known as a type II single-pass transmembrane protein that regulates bidirectional scrambling of phospholipids across lipid bilayer in a Ca^2+^-dependent and ATP-independent manner [38]. But hPLSCR1 also binds to a specific sequence in the 5′-promoter region of the inositol 1,4,5-triphosphate receptor type 1 gene (IP3R1) and acts like a transcriptional activator that eventually regulates cell proliferation and maturation [39]. Furthermore, it is known that hPLSCR1 also exhibits Mg^2+^-dependent DNase activity with an optimum at 37 °C and pH 8.5 [34]. Likewise, human apurinic/apyrimidinic nuclease APEX1 is a key enzyme for base excision repair pathway involved in the recognition and repair of the abasic site, one of the most common DNA damages, and hence plays a critical role in genome maintenance [40]. However, studies also reported that this protein also functions as a reduction-oxidation (redox) factor and thus regulates many transcription factors [41].

### 2.5. AtBBD1 and AtBBD2 Have Non-Substrate-Specific RNase Activity

**Figure 7:**
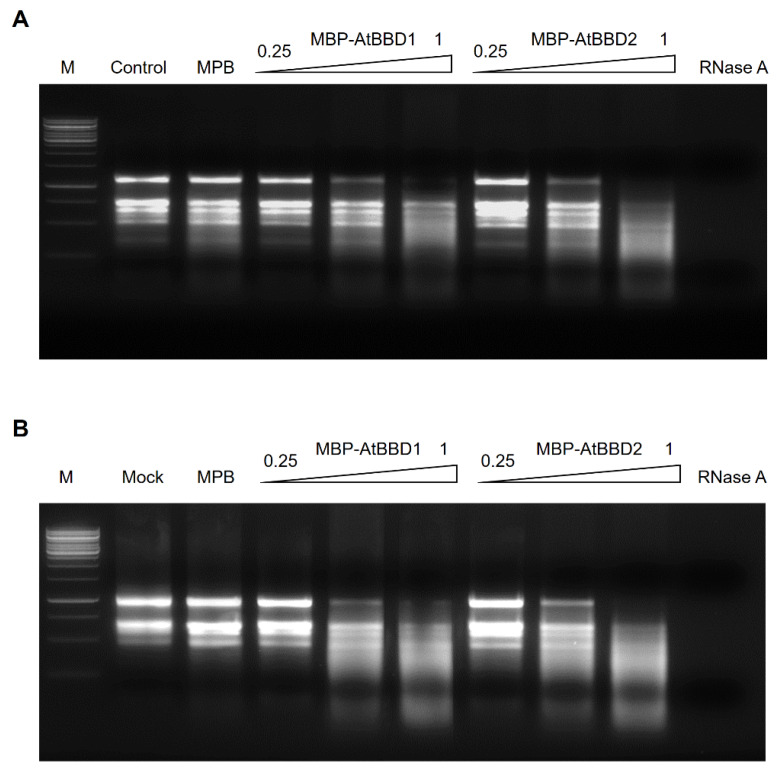
RNase activity of the MBP-AtBBD1 and MBP-AtBBD2 proteins. (**A**) *Arabidopsis thaliana* total RNA (2 μg) was incubated with 0.25 to 1 μg of purified protein at 37 °C for 2 h. (**B**) *Oryza sativa* total RNA (2 μg) was incubated with 0.25 to 1 μg of purified protein at 37 °C for 2 h. The degraded RNA products were loaded onto a 1.0% agarose gel. Lanes M, DNA marker; Mock, without any protein. One unit of commercial RNase A was used as a positive control.

We also analyzed the in vitro RNase activity of AtBBD1 and AtBBD2 using total RNAs extracted from *Arabidopsis* and rice as the substrates (Figure 7A,B). While MBP, a negative control, did not produce any visible degradation of RNA, MBP-AtBBD1 and MBP-AtBBD2 degraded *Arabidopsis* RNA (Figure 7A) and *Oryza sativa* RNA (Figure 7B) as indicated by smearing in a concentration-dependent manner, as OmBBD does. Unlike OmBBD, however, the RNase activity of AtBBD1 was abolished when it was incubated at 95 °C (Figure S3). These data indicate that AtBBD1 and AtBBD2 possess non-substrate-specific RNase activity.

### 2.6. AtBBD1 and AtBBD2 Each form a Homodimer and the HCR Domain is Possibly Crucial for Dimerization

**Figure 8:**
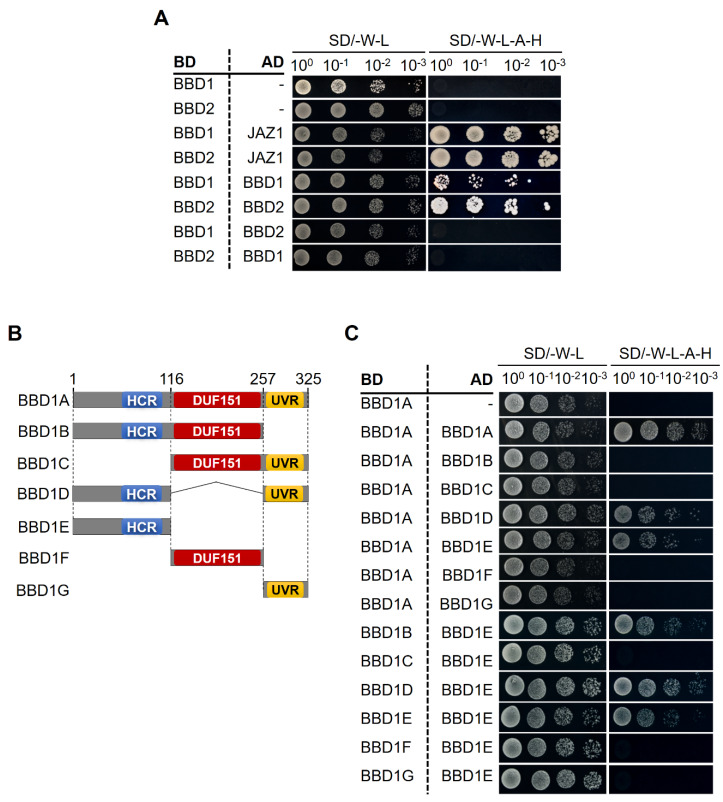
Yeast two-hybrid assay. (**A**) The homomeric interaction of AtBBD1 and AtBBD2. BD and AD represent empty vectors of pGBKT7 and pGADT7, respectively. BD-BBD1 + AD-JAZ1 and BD-BBD2 + AD-JAZ1 are positive controls; BD-BBD1 + AD and BD-BBD2 + AD are negative controls. (**B**) A schematic representation of the domain deletion series of AtBBD1. Numbers indicate amino acid residues. (**C**) Domain deletion series of AtBBD1 for homomeric domain identification. Full length and truncated versions of AtBBD1 proteins containing HCR, DUF151 and UVR individually or in a combination were used.

To check if AtBBD1 and AtBBD2 each form a dimer, we performed a yeast two-hybrid assay and confirmed that both AtBBD1 and AtBBD2 form a homodimer. However, these two proteins did not form a heterodimer, even though they have very high sequence identity (80.31%) (Figure 8A). Because these proteins are comprised of three distinct domains like OmBBD, to identify which domain is responsible for the homodimerization, we performed a yeast two-hybrid assay with a domain deletion series of AtBBD1 (Figure 8C). Initially, we confirmed that none of our constructs showed any auto-activation (Figure S4). Our data demonstrated that the HCR domain is essential to form the homodimerization (Figure 8C) and were consistent with the result of Seo et al. [29]. On the other hand, TM0160 and Rv1829 each form a homodimer by means of DUF151 domain. Crystal structure of TM0160 suggested that Asp115 and His58 of one monomer and Thr57 of another monomer form a putative catalytic triad to make a putative active site pocket [42]. The amino acid sequence alignment of 262 seed proteins containing DUF151 domain obtained from the Pfam database showed that aspartic acid at position 163, which corresponds positions 226 and 230, respectively, in AtBBD1 and AtBBD2, is 100% conserved in all species aligned (data not shown). Although we found that HCR domain is more likely to be responsible for homodimer formation in the case of AtBBD1, we suspect that a conserved aspartic acid could be important for formation of an active site in the DUF151-containing proteins including AtBBD1 and AtBBD2, but further studies are required for it to be elucidated. Moreover, extensive in planta experiments are also needed to characterize the physiological function of AtBBD1 and AtBBD2.

## 3. Materials and Methods

### 3.1. Gene Cloning and Construction of Expression Vector

The coding region of *AtBBD1* and *AtBBD2* was amplified from *Arabidopsis* cDNA using the cloning primers 5′-CGGAATTCATGAGATCGGTTCAAGCACCAGTTG-3′ (*Eco*RI site underlined) and 5′-CGCGGATCCTCATGTGTATTTCCTCAAGTTACGTTTG GC-3′ (*Bam*HI site underlined), and 5′-CGGAATTCATGAGGTCGCTTCAAGCACCG-3′ (*Eco*RI site underlined) and 5′-CGGGATCCTCATGTGTATTTCCTTAATTTGCGTTTTGCCTG-3′ (*Bam*HI site underlined), respectively. Around 10 ng of cDNA were used in a 50-μL reaction containing PrimeSTAR™ HS DNA Polymerase (Takara Bio Inc., Kusatsu, Japan) and amplified using the following PCR conditions: initial denaturation at 98 °C for 5 min, followed by 30 cycles of denaturation at 98 °C for 10 s, primer annealing at 58 °C for 4 s and extension at 72 °C for 60 s, and final extension at 72 °C for 5 min. The PCR products were cleaned up, digested with *Eco*RI/*Bam*HI and cloned into the corresponding site of the pMAL-C2X vector (New England Biolabs, Beverly, MA, USA). The empty pMAL-C2X vector, pMAL-AtBBD1 and pMAL-AtBBD2 constructs were transformed separately into *E. coli* DH5α (Thermo Fisher Scientific, Waltham, MA, USA) competent cells. The results of cloning were determined by DNA sequence analysis (Bionics, Seoul, South Korea). After sequence confirmation, the empty pMAL-C2X vector, pMAL-AtBBD1 and pMAL-AtBBD2 constructs were transformed separately into *E. coli* Rosetta™ (DE3) (Novagen, Madison, WI, USA) competent cells for protein expression and purification.

### 3.2. Expression and Purification of MBP-BBD1 and MBP-BBD2

The transformed *E. coli* Rosetta™ (DE3) harboring pMAL-C2X, pMAL-AtBBD1 and pMAL-AtBBD2 were grown overnight in LB media containing carbenicillin (50 mg/L) and chloramphenicol (25 mg/L) at 37 °C. One percent of the overnight culture was transferred into a fresh LB medium containing the same antibiotics and grown at 37 °C to OD (optical density)_600_ = 0.6. After adding isoprophyl β-d-1-thiogalactopyranoside (IPTG) to a final concentration of 0.5 mM, the culture was incubated for an additional 20 to 24 h at 18 °C and harvested by centrifugation (5000× *g*, 10 min, 4 °C). The pellet was resuspended in 25 mL of ice-cold resuspension buffer (50 mM Tris-HCl, 500 mM NaCl, pH 7.4, 1 mM dithiothreitol (DTT), 0.5 mM phenylmethylsulfonyl fluoride (PMSF) and a tablet of the cOmplete™ Protease Inhibitor Cocktail (Roche, Basel, Switzerland) and 1% Triton X-100). The resuspended cells were lysed by sonication and kept on ice for 30 min. The cell lysate was centrifuged at 25,000× *g* for 30 min at 4 °C. The supernatant was filtered through a 0.45 μM filter and transferred to a fresh 50 mL falcon tube. For protein purification, the amylose resin (New England Biolabs, Beverly, MA) equilibrated with resuspension buffer was added to the filtered supernatant and then incubated with gentle rocking for 30 min at 4 °C. The proteins were then loaded on a column (Poly-Prep® Chromatography Column, Bio-Rad Laboratories, Hercules, CA, USA) and the unbound proteins were removed with washing buffer (50 mM Tris-HCl, 200 mM NaCl, pH 7.4, 0.5 mM DTT) three times. The proteins were eluted with 5 mL of elution buffer (washing buffer containing 10 mM maltose) four times. The eluted fractions were further concentrated by Amicon centrifugal filters (50 kDa cut-off) (Millipore, Burlington, MA, USA). Samples of eluted proteins (15 μL) were electrophoresed on 12% SDS-polyacrylamide gels (SDS-PAGE) and visualized with Coomassie blue staining. Bradford reagent (Sigma-Aldrich, St. Louis, MO, USA) was used to quantify the protein concentration.

### 3.3. Nuclease Activity of Recombinant BBD1 and BBD2

Nuclease activity assay was performed with DNA and RNA substrates using purified recombinant BBD1 and BBD2 proteins. For DNase activity, 0.5 μg of salmon sperm DNA, *Arabidopsis* genomic DNA, circular and linearized pMAL-C2X plasmids were incubated with 1 μg of the purified MBP, MBP-BBD1 and MBP-BBD2 proteins in a 20-μL nuclease reaction buffer (50 mM Tris–HCl, pH 8.0, 250 mM NaCl and 0.5 mM dithiothreitol) at 37 °C for 3 h. Likewise, for RNase activity, 2 μg of *Arabidopsis* total RNA was incubated with an increasing concentration of the purified proteins ranging from 0.25 to 1 μg in a 20-μL reaction buffer at 37 °C for 3 h. The reaction products were separated by electrophoresis on 1% agarose gel with ethidium bromide (0.5 μg/mL). To rule out the possibility that the nuclease activity was derived from any other external DNases and RNases and that the MBP tag has no nuclease activity, we used buffer and MBP protein as negative control in this assay. One unit of commercial DNase I (Invitogen, Carlsbad, CA, USA) and RNase A (Sigma-Aldrich, St. Louis, MO, USA) were used as positive controls.

The effect of temperature and pH on nuclease activity was measured according to the Kunitz assay. One Kunitz unit was defined as the amount of enzyme added to 1 mg/mL of DNA that causes an increase in absorbance of 0.001 per minute. The increase in absorbance is due to the release of free nucleotides upon degradation of polymerized DNA [43]. Kunitz assays were performed with 10 pmol/μL of purified recombinant MBP-BBD1 and MBP-BBD2 protein and 0.01-mg/mL *Arabidospis* genomic DNA in the nuclease reaction buffer for 1 h at a series of temperature and pHs. The DNA substrate without a purified protein was used as a negative control. After incubation, the reaction was stopped by adding an equal volume of 5% ice-cold perchloric acid, kept on ice for 10 min and then centrifuged at 7500× *g* for 5 min. The absorbance of supernatant was measured at 260 nm using a Synergy H1 spectrophotometer (BioTek Instruments Inc., Winooski, VT, USA). The difference in A_260_ between the control (DNA without proteins) and a sample (DNA with purified proteins) was measured and the enzyme activity was quantified by subtracting the control A_260_ value from sample A_260_ value. The relative enzyme activity was calculated by dividing each enzyme activity at a temperature or pH value by the highest enzyme activity at a temperature or pH followed by multiplication by 100. The highest activity was defined as 100% and results were expressed as relative activity in percentage.

The effect of cofactors on DNase activity was determined using 0.1 μg *Arabidopsis* gDNA was incubated with 1 μg of the purified MBP-BBD1 and MBP-BBD2 proteins in a 20-μL nuclease reaction buffer (50 mM Tris–HCl, pH 8.0, 250 mM NaCl and 0.5 mM dithiothreitol) at 37 °C for 1 h in the presence of 10 mM of different divalent cations (CaCl_2_, MgCl_2_, MnCl_2_ or ZnCl_2_). All reactions were terminated by the addition of 5 μL 5X termination buffer (2.5X SDS, 500 mM EDTA, 25% glycerol and 1% β-mercaptoethanol) and incubated at 80 °C for 10 min. After incubation, the reaction tube was centrifuged at 7500× *g* for 10 min and was subjected to 1% agarose gel electrophoresis containing ethidium bromide (0.5 μg/mL). The intensity of degraded DNA bands in the gels was quantified using ImageJ software (version 1.8.0_112). The relative value of degraded DNA substrate was calculated as the relative ratio of the band intensity in each lane to the band intensity in the control lane (no enzyme).

### 3.4. Construction of Phylogenetic Tree and Sequence Analysis

Homology searches were performed with the sequences in the GenBank database using the Protein BLAST program. The sequences of putative DUF151 domain-containing proteins from representative species were collected from the Pfam database (https://pfam.xfam.org/family/PF02577), SMART architecture analysis (http://smart.embl-heidelberg.de) and previous reports [15,29]. A phylogenetic tree was generated using the neighbor-joining method using the MEGA-X program (version 10.1.7) (https://www.megasoftware.net/). The domain architecture was constructed using the NCBI Batch CD-search (https://www.ncbi.nlm.nih.gov/Structure/bwrpsb/bwrpsb.cgi) and retouched with Adobe Illustrator. Multiple sequence alignment was performed using the GeneDoc program (version 2.7). The percentage similarities among proteins were analyzed using Clustal Omega (http://www.clustal.org/omega/) (version 1.2.2).

### 3.5. Yeast Two-Hybrid

For protein–protein interactions, the yeast two-hybrid MATCHMAKER system (Clontech, Mountain View, CA, USA) was used according to the manufacturer’s protocols. The open reading frames of *AtBBD1* and *AtBBD2* were cloned in frame into the pGBKT7 vector containing the GAL4 DNA binding domain and the pGADT7 vector containing the GAL4 activation domain. The yeast strain AH109 harboring the *LacZ*, *His*, and *Ade* reporter genes was used for transformation with each vector. The transformed yeast cells were serially diluted and dropped onto the synthetic dropout (SD) medium lacking leucine, tryptophan, adenine and histidine (SD/-L-W-A-H) or lacking leucine and tryptophan (SD/-L-H). The yeast cells were incubated at 30 °C and the growth of the cells was observed after five days.

## Supplementary Materials

The following are available online, Table S1: DUF151-containing proteins used in this study, Figure S1: SDS-PAGE gels of the His-BBD1, His-BBD2, GST-BBD1 and GST-BBD2 proteins in pellets and soluble fractions, Figure S2: Solubilization of AtBBD1 and AtBBD2 proteins, Figure S3: RNase activity assay of the native and boiled MBP-BBD1 protein, Figure S4: Yeast auto-activation test of the full length and the domain deletion series of AtBBD1.
