# Supplementary material for: Phylogenetic Analysis and In Vitro Bifunctional Nuclease Assay of Arabidopsis BBD1 and BBD2
Source: Molecules. 2020 May 6;25(9):2169. doi: 10.3390/molecules25092169 (PMC7249048; doi:10.3390/molecules25092169)
Supplement: Supplementary file 1 [file molecules-25-02169-s001.pdf]

<Supplementary Materials>

Table S1. DUF151-containing proteins used in this study.

Name in the Phylogenetic Tree	Uniprot ID / NCBI Ref. No.	Superkingdom (Domain)	Kingdom / Phylum	Organism
AtBBD1	Q9FWS6	**Eukarya**	Viridiplantae	*Arabidopsis thaliana*
AlBBD1	Q9FKX7	”	”	*Arabidopsis lyrata*
BrBBD1	M4CVE4	”	”	*Brassica rapa*
BnBBD1	A0A078JL75	”	”	*Brassica napus*
AtBBD2	Q93VH2	”	”	*Arabidopsis thaliana*
AlBBD2	D7KI45	”	”	*Arabidopsis lyrata*
BrBBD2	M4EAE7	”	”	*Brassica rapa*
BnBBD2	A0A078GW62	”	”	*Brassica napus*
MtBBD2	A0A072VDT6	”	”	*Medicago tranculata*
GmBBD1	K7M5E0	”	”	*Glycin max*
VvBBD	E0CQH3	”	”	*Vitis vinifera*
NtBBD1	A0A1S4AP95	”	”	*Nicotiana tabacum*
OsiBBD2	A2YUD2	”	”	*Oryza sativa indica*
OsjBBD2	XP_015650236.1	”	”	*Oryza sativa japonica*
SbBBD2	XM_021464813.1	”	”	*Sorghum bicolor*
ZmBBD3	NP_001158999.1	”	”	*Zea mays*
ZmBBD1	A0A1D6MW07	”	”	”

ZmBBD2	B6TCQ8	”	”	”
SbBBD1	XM_021446529.1	”	”	*Sorghum bicolor*
OsiBBD1	B8A8D2	”	”	*Oryza sativa indica*
OmBBD	Q09LL3	”	”	*Oryza minuta*
OsjBBD1	Q5N8J3	”	”	*Oryza sativa japonica*
PpBBD1	XM_024501638.1	”	”	*Physcomitrella patens*
PpBBD2	XM_024526960.1	”	”	”
SmBBD	D8RQB2	”	”	*Selaginella moellendorffii*
NtBBD2	A0A1S4ABC4	”	”	*Nicotiana tabacum*
AtBBD3	Q8GWL4	”	”	*Arabidopsis thaliana*
GmBBD2	A0A0R0LBR6	”	”	*Glycin max*
CrBBD	XM_001698860.1	”	”	*Chlamydomonas reinhardtii*
Hs_ Q9HMD3	Q9HMD3	**Archaea**	Euryarchaeota	*Halobacterium Salinarum*
Np_ A0A1U7ETY5	A0A1U7ETY5	”	”	*Natronomonas pharaonis*
Ea_ A0A2D7UJM4	A0A2D7UJM4	”	”	*Euryarchaeota archaeon*
Ea_ A0A2E7AYD6	A0A2E7AYD6	”	”	”
Pa_ Q8ZT14	Q8ZT14	”	Crenarchaeota	*Pyrobaculum aerophilum*
Ml_ A0A0H3N0F9	A0A0H3N0F9	**Bacteria**	Actinobacteria	*Mycobacterium leprae*
Mt_ P9WLR4	P9WLR4	”	”	*Mycobacterium tuberculosis*
Sc_ H1Q7W1	H1Q7W1	”	”	*Streptomyces coelicoflavus*
Fg_ A0A1M5PP02	A0A1M5PP02	”	Bacteroidetes	*Flavobacterium granuli*

Bu_ A0A078RZV9	A0A078RZV9	”	”	*Bacteroides uniformis*
Bf_ A0A081U9G6	A0A081U9G6	”	”	*Bacteroides fragilis*
Bt_ R9H9M8	R9H9M8	”	”	*Bacteroides thetaiotaomicron*
Cc_ Q822P8	Q822P8	”	Chlamydiae	*Chlamydophila caviae*
Cp_ Q9Z943	Q9Z943	”	”	*Chlamydia pneumoniae*
Ct_ O84214	O84214	”	”	*Chlamydia trachomatis*
Cl_ B3EFD8	B3EFD8	”	Chlorobi	*Chlorobium limicola*
Chp_ B3ELR6	B3ELR6	”	”	*Chlorobium phaeobacteroides*
Tm_ Q9WY07	Q9WY07	”	Thermotogae	*Thermotoga maritima*
Tn_ Q5CBM7	Q5CBM7	”	”	*Thermotoga naphthophila*

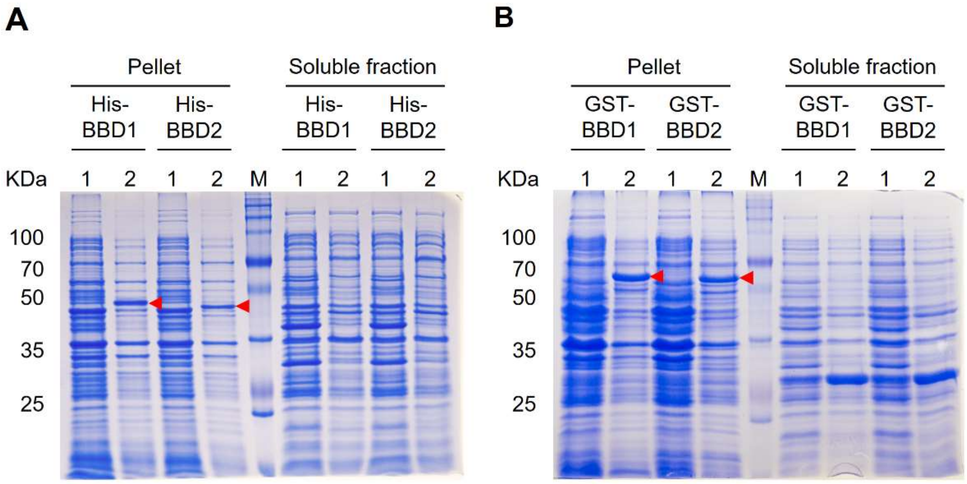

Figure S1. SDS-PAGE gels of the His-BBD1, His-BBD2, GST-BBD1 and GST-BBD2 proteins in pellets and soluble fractions. Total proteins were electrophoresed on a 12% SDS-PAGE gel and stained with Coomassie blue R-250. Lane M, protein marker; 1, un-induced cells; 2, induced cells. Target proteins are indicated by red arrowheads. (A) SDS-PAGE of the His-BBD1 and His-BBD2 proteins. (B) SDS-PAGE of the GST-BBD1 and GST-BBD2 proteins.

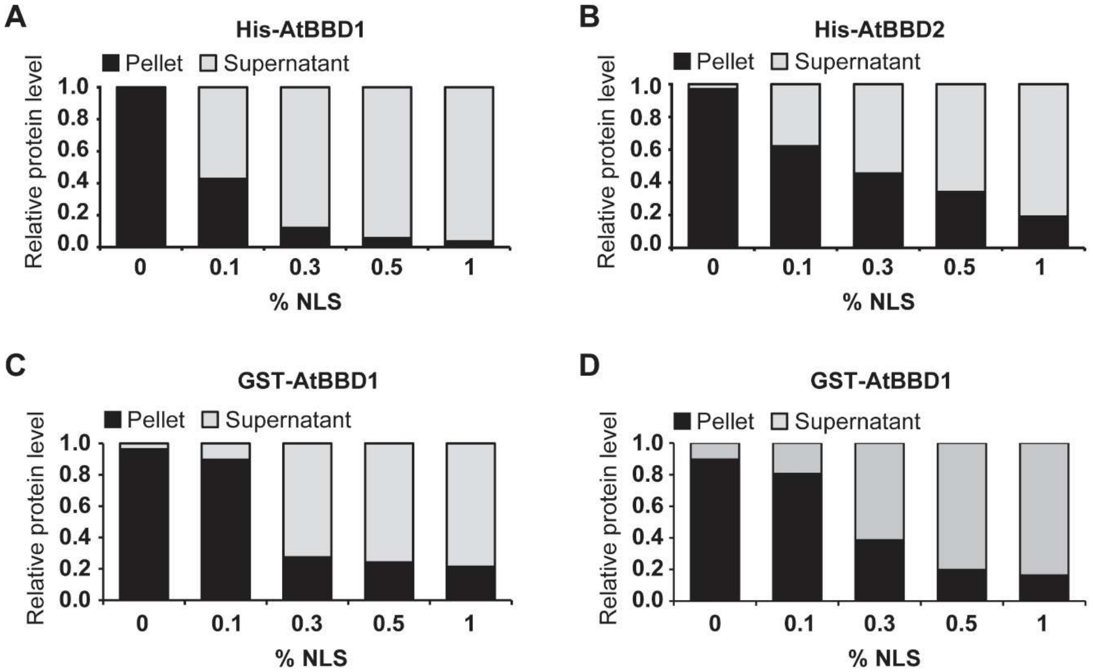

Figure S2. Solubilization of AtBBD1 and AtBBD2 proteins. His-AtBBD1, His-AtBBD2, GST-AtBBD1 and GST-AtBBD2 proteins were solubilized using 0.1, 0.3, 0.5 and 1% non-denaturing alkylanionic detergent N-lauroylsarcosine sodium salt (NLS). Quantification of the solubility of (A) His-AtBBD1, (B) His-AtBBD2, (C) GST-AtBBD1 and (D) GST-AtBBD2. Band intensity in each fraction was determined by ImageJ software and the relative values of the amount of proteins in the pellet and supernatant factions were expressed as the ratio of the band intensity in each fraction to those in the sum of pellet and supernatant fractions (Sum = 1).

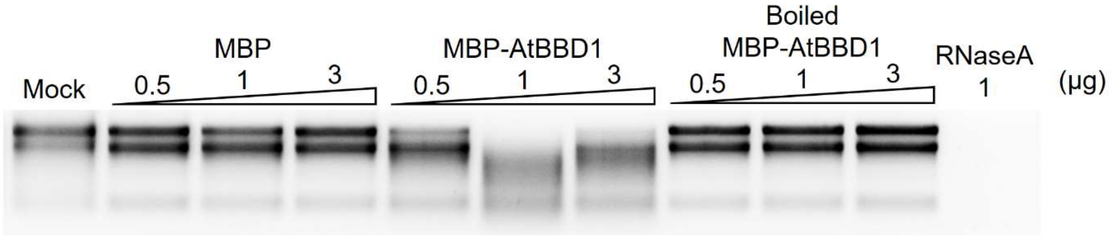

Figure S3. RNase activity assay of the native and boiled MBP-BBD1 protein. *Arabidopisis* total RNA (2 μg) was incubated with 0.25 to 3 μg of purified proteins at 37° C for 1 h. The degraded RNA products were loaded onto a 1.0% agarose gel. Lanes M, DNA marker; EB, elution buffer. One unit of RNase A was used as a positive control.

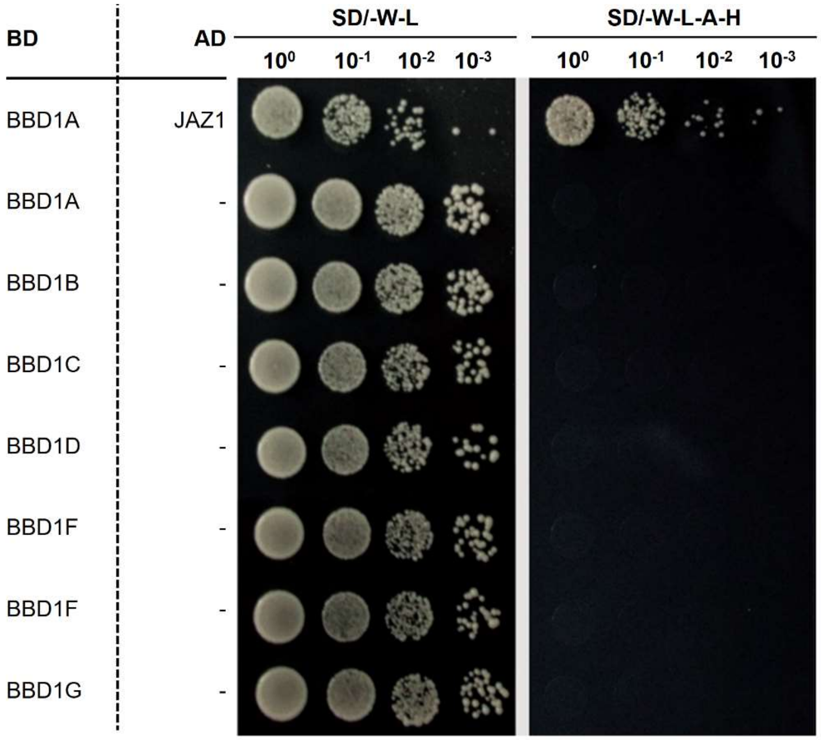

Figure S4. Yeast auto-activation test of the full length and the domain deletion series of AtBBD1. BD and AD represent the empty vector of pGBKT7 and pGADT7, respectively. BD-BBD1A + AD-JAZ1 is a positive control.
